# A translational framework for public health research

**DOI:** 10.1186/1471-2458-9-116

**Published:** 2009-04-28

**Authors:** David Ogilvie, Peter Craig, Simon Griffin, Sally Macintyre, Nicholas J Wareham

**Affiliations:** 1 Medical Research Council Epidemiology Unit and Centre for Diet and Activity Research (CEDAR), Cambridge, UK; 2 Medical Research Council Population Health Sciences Research Network, Glasgow, UK; 3 Medical Research Council Social and Public Health Sciences Unit, Glasgow, UK

## Abstract

**Background:**

The paradigm of translational medicine that underpins frameworks such as the Cooksey report on the funding of health research does not adequately reflect the complex reality of the public health environment. We therefore outline a translational framework for public health research.

**Discussion:**

Our framework redefines the objective of translation from that of institutionalising effective interventions to that of improving population health by influencing both individual and collective determinants of health. It incorporates epidemiological perspectives with those of the social sciences, recognising that many types of research may contribute to the shaping of policy, practice and future research. It also identifies a pivotal role for evidence synthesis and the importance of non-linear and intersectoral interfaces with the public realm.

**Summary:**

We propose a research agenda to advance the field and argue that resources for 'applied' or 'translational' public health research should be deployed across the framework, not reserved for 'dissemination' or 'implementation'.

## Background

The translation of health research is increasingly regarded as important – not only in the UK but also across Europe and North America – in order to maximise the population health benefits of investment in research and health care delivery [1]. However, 'translation' and 'translational research' mean different things to different people. The Canadian Institutes of Health Research (CIHR) define 'knowledge translation' in terms of exchange, synthesis, dialogue and interaction between researchers and users – a 'radically different' model from the unidirectional flow of knowledge sometimes implied by terms such as 'dissemination' or 'knowledge transfer' [2]. It is also recognised that many contemporary health challenges require a more fundamental and wide-ranging societal response than those that can be offered through established systems of delivering health care [3]. This is the domain of public health, the nature and scope of which is not universally understood [4].

In this paper, we therefore propose a translational framework for public health research. Although our framework is presented in the context of a case study of the current situation in the UK, the issues we address are general and equally applicable to an international audience.

### Translational research and clinical medicine

The Cooksey report, whose recommendations were accepted by the Treasury in December 2006, defined a new framework for the funding of health research in the UK [5-7]. Among other things, the report recommended devoting a greater share of funding to 'translational' research and the establishment of a board for translational medicine under the auspices of the new cross-cutting Office for Strategic Coordination of Health Research (OSCHR).

**Figure 1:**
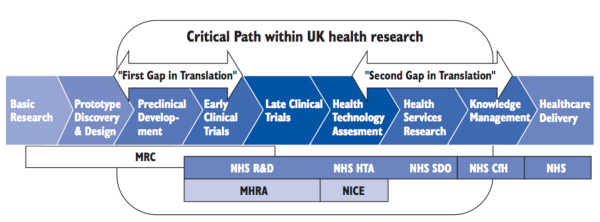
**Pathway for translation of health research into healthcare improvement**. *Source: A review of UK health research funding *(the Cooksey report) [5]. ^© ^Crown copyright 2006. Reproduced with permission. Blue boxes below parts of pathway correspond to specific responsibilities of public sector bodies supporting research. MRC: Medical Research Council. NHS R&D: National Health Service Research and Development. NHS HTA: NHS Health Technology Assessment Programme. NHS SDO: Service and Delivery Organisation research programme. NHS CfH: Connecting for Health. Light blue boxes below parts of pathway correspond to the specific responsibilites of statutory regulatory agencies. MHRA: Medicines and Healthcare products Regulatory Agency. NICE: National Institute for Health and Clinical Excellence.

The report describes a 'pathway for translation of health research into healthcare improvement' and identifies two principal gaps in that pathway: 'the translation of basic and clinical research into ideas and products', and 'introducing those ideas and products into clinical practice' (Figure 1). Bridging the first gap (sometimes labelled as T1) [1] involves preclinical development and early clinical trials, whereas bridging the second (T2) involves health technology assessment, health services research and knowledge management [5]. Like the influential 'roadmap' developed by the National Institutes of Health (NIH) in the United States [8], the pathway described is clearly based on the process of drug development, whose aims are to turn the findings of 'basic science' into a new drug and to incorporate the use of the new drug into routine clinical practice for the benefit of individual patients [9,10].

### Towards a translational framework for public health research

Stakeholders and commentators have generally supported Cooksey's recommendation to increase investment in translational research. However, it is unclear whether the linear, basic-to-applied, 'translational medicine' paradigm is applicable to public health (or, indeed, to some other related fields such as health care policy). In the case of obesity, for example, despite the volume of relevant basic science in genetics, physiology, pharmacology and the behavioural sciences, the obvious translational outputs from this research – such as weight-loss drugs, or campaigns to persuade the population to eat less and exercise more – have not reversed the rising trend in the prevalence of obesity in the general population and appear unlikely to do so [11,12]. The principle that public health research is important and needs to be translated does not appear to be in dispute. However, two critical questions require to be addressed: what constitutes the public health research to be translated, and how might that translation be carried out effectively? Recent critiques of the NIH roadmap have begun to address this problem, but have remained resolutely focused on the question of how to improve clinical practice [8,13].

We aimed to develop a translational framework for public health research to help define and inform future work in this field. Our aim was to map the territory, the 'big picture' to which public health research should relate, rather than to prescribe a particular route to be followed. We intended to encompass both the 'iterative, bidirectional circuitry of scientific discovery' [14] by which public health research and public health action may influence each other, and the research that may underpin, inform or illuminate these interactions. We derived the framework in a series of logical steps starting from the translational pathway described in the Cooksey report [5], aiming to balance a desire for a simple (intelligible) model with the need to reflect the complexity of the public health environment. We drew on the definitions of public health and public health sciences used in the Acheson and Frankel reports respectively and the circular model of 'diabetes translation' described by Narayan and colleagues (Table 1) [15-18].

**Table 1:** Starting points: selected definitions

**Construct**	**Definition**
Public health	'the science and art of preventing disease, prolonging life, and promoting health through organized efforts of society' (Acheson report, 1998) [15]
Public health sciences	'Effective public health actions are based on scientifically derived information about factors influencing health and disease and about effective interventions to change behaviour at the level of the individual, the family, the community or wider society [...] The public health sciences are essential to further our understanding of the relative importance of environmental, lifestyle and genetic causes of disease[,] to identify strategies to improve the wellbeing of the population and to evaluate their impact' (Frankel report, 2004) [16]
Translational research	'comprehensive applied research that strives to translate the available knowledge and make it useful...' (Narayan et al, 2000) [17]

**Figure 2:**
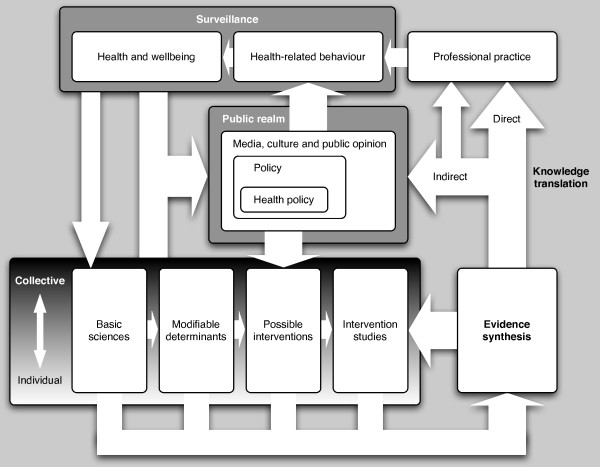
**Translational framework for public health research**.

The framework is shown in Figure 2 and its key features are summarised in Table 2. Four case studies (Table 3, Table 4, Table 5 and Table 6) illustrate the limitations of the linear translational medicine pathway and the importance of considering this wider framework.

**Table 2:** Key differences between the translational framework for public health research and the linear translational medicine pathway

**Characteristics of the translational framework for public health research**

Redefines the endpoint from that of institutionalising effective interventions to that of improving population health

Incorporates the epidemiological traditions of population health surveillance and the identification of modifiable risk factors

Reflects a spectrum of determinants of health from the individual to the collective level and a corresponding spectrum of levels of intervention

Embraces a wide range of biomedical, social and environmental 'basic sciences' that have roles throughout the framework, not merely in supplying knowledge to be implemented

Identifies a pivotal role for thoughtful and inclusive evidence synthesis

Describes the iterative and bidirectional processes by which public health research and public health action may influence each other

Recognises the non-linear and intersectoral interfaces with the public realm where decisions that influence population health are made

**Table 3:** Dietary salt and blood pressure

**Case study**

A dose-response relationship between dietary salt intake and blood pressure has been consistently demonstrated in animal studies and in ecological, cohort and intervention studies in humans. A recent randomised controlled trial has also shown that dietary and behavioural counselling to limit salt intake reduces the incidence of 'hard' cardiovascular endpoints, thus surely fulfilling any reasonable definition of an evidence-based public health intervention. The linear model of translation suggests that all that remains is for 'sodium reduction interventions' of this kind to be implemented as widely as possible [20,21].

However, an estimated 80% of dietary salt intake in Westernised countries comes from bread and processed foods rather than from discretionary use. Even if it were feasible to roll out intensive counselling across the population, shifting the population distribution of salt intake is therefore more likely to depend on changing the composition of processed foods. The greatest potential for translation into population health improvement may therefore lie not in disseminating and implementing a 'proven' intervention but in using other, predominantly epidemiological evidence to influence policymakers and the non-statutory corporate social responsibilities of food manufacturers. It may not be possible to demonstrate the population-level effectiveness (or otherwise) of regulatory interventions on food labelling or the salt content of processed foods until policymakers, somewhere, decide to intervene in this way as a 'natural experiment'; the effects could then be evaluated through enhanced population dietary and health surveillance [36].

**Table 4:** Sleeping position and sudden infant death syndrome

**Case study**

'Back to Sleep' campaigns to discourage the prone sleeping position are credited with having reduced the incidence of sudden infant death syndrome (SIDS) by 50–70%. The success of these campaigns reflects the effective translation of the findings of research in pathology and epidemiology into a comparatively simple intervention that was then effectively disseminated to health care professionals and parents.

However, the linear translational medicine pathway neither accounts for this success nor offers an obvious route to further reducing the impact of SIDS on the population. The case for 'Back to Sleep' was based not on clinical trials showing that the proposed intervention was effective, but on epidemiological evidence that the prone sleeping position was a risk factor for SIDS. The value of synthesising non-trial evidence is illustrated by the retrospective finding that this association could have been established by a meta-analysis of case-control studies as early as 1970, whereas many textbooks continued to recommend the prone sleeping position until the late 1980s. It has only been possible to demonstrate the effectiveness of the intervention after its widespread introduction and by using observational study designs. Meanwhile, continuing surveillance and observational epidemiology highlight possible side-effects such as an increase in plagiocephaly and show that SIDS is increasingly associated with deprivation, suggesting a need for action elsewhere in the public realm to reduce the risk among babies born into poor families [22-25].

**Table 5:** Human papillomavirus vaccine for cervical cancer

**Case study**

Human papillomovirus (HPV) vaccine can be used to protect adolescent girls against cervical cancer. Current calls to introduce an immunisation programme reflect the cumulation of evidence from aetiological epidemiology, which has identified HPV as a necessary cause of most cervical cancers, and translational medicine, which has produced a vaccine and shown it to be safe and effective in randomised controlled trials. The linear model of translation suggests that all that remains is for an immunisation programme to be implemented in primary care.

However, the UK experience of other recent translational activities in this field illustrates how these may have queered the pitch for new vaccines. The findings of one single, small and unreplicated study [37] were interpreted as showing that the measles, mumps and rubella (MMR) vaccine may cause autism and were deliberately released into the public realm where they were amplified and disseminated by the mass media, resulting in a marked decrease in MMR coverage to a low of 79% in England in 2003 [26]. This unintentional translational process was much more effective in changing population health-related behaviour (vaccine uptake) than was the subsequent systematic synthesis of epidemiological evidence [27]. It also directly produced a change in professional practice (the introduction of single-vaccine clinics) without this ever being recommended in clinical guidelines. The successful translation of the potential offered by the HPV vaccine into actual population health improvement will therefore depend, among other things, on winning the argument in the public realm that the benefits of routine immunisation outweigh the harms, and in particular that it is appropriate to immunise girls against a sexually transmitted infection before they become sexually active. Meanwhile, parents' attitudes and reactions to information about vaccines remains an active area of research that could be described as 'basic science' – in the sense that it investigates the causes of health-related behaviour – but is also clearly crucial to the effective translation of future advances in this field [28-31].

**Table 6:** NICE guidance on physical activity and the environment

**Case study**

The National Institute for Health and Clinical Excellence (NICE) provides evidence-based guidance for clinical practice in the National Health Service (NHS) in England. Each piece of guidance is based on the systematic review of evidence for the effectiveness and cost-effectiveness of interventions and is subsequently translated into a set of implementation materials. In principle, the NICE process therefore fits neatly into the 'health technology assessment' component of the linear translational medicine pathway.

However, NICE's remit was expanded in 2005 to include public health, and its recent guidance on physical activity and the environment illustrates the need for a more inclusive translational framework. Most intervention studies reviewed for this guidance were of comparatively low quality and few demonstrated unequivocal changes in physical activity. However, rather than conclude that the evidence was insufficient, the programme development group drew on other types of evidence admissible under NICE procedures – including evidence about environmental correlates of physical activity, and interdisciplinary expert consensus – to make constructive recommendations based on a more inclusive approach to evidence synthesis. Most recommendations were intended for recipients outside the NHS such as transport planners, who have not previously been the target of NICE guidance and are under no obligation to take account of it. The successful implementation of this guidance is therefore likely to depend more on the 'indirect insinuation' of the recommendations into the practice of those working outside the health sector, perhaps by articulating an additional, public health case for interventions primarily motivated by other aims such as reducing traffic [35].

## Discussion

### Characteristics of the framework

Our framework departs from the linear translational medicine pathway described by Cooksey in several important ways.

#### An epidemiological perspective on the process and the endpoint

In contrast to the laboratory sciences that underpin translational medicine, epidemiology is the discipline that has traditionally been considered the core basic science for public health. In the classical epidemiological paradigm, descriptive (hypothesis-generating) studies lead to analytical (hypothesis-testing) studies based on cohort and case-control designs that identify putative risk factors for disease. Where strong evidence can be found for a causal relationship (e.g. according to the viewpoints enumerated by Bradford Hill such as the importance of temporal, dose-response and reversible associations) [19], these risk factors inform the selection, development and evaluation of putative interventions to influence those risk factors – either directly, or by targeting determinants of individuals' health-related behaviour that may in turn influence those risk factors (Table 3) [20,21].

The translational medicine pathway assumes that the desired endpoint is the incorporation of an intervention that has been declared effective into routine clinical practice [14]. However, the Acheson definition sees the objectives of public health in much wider terms. Changes in practice may well be necessary, but cannot possibly be considered sufficient as an endpoint for public health purposes. Our framework therefore redefines the endpoint as population health improvement, whether ascertained as changes in health-related behaviour or other risk factors (in the shorter term), wellbeing or quality of life, or 'hard' morbidity or mortality endpoints (in the longer term). This implies a need for a feedback loop whereby population health surveillance data contribute to the descriptive epidemiology of the conditions under surveillance and their risk factors (Table 4) [22-25]. It also acts as a counterweight to a clinically-oriented understanding of public health that sees public health practice as mainly concerned with optimising health care rather than with primary or primordial prevention [17,18].

#### A social perspective on the basic sciences of public health

Most epidemiological research reflects an individual-level perspective on health and its determinants. In contrast, our framework reflects a spectrum of determinants of health from modifiable risk factors (at the individual level) to the social determinants of health (at the collective level). Logically, it also embraces a corresponding spectrum of levels of intervention and of relevant 'underpinning' or 'basic' sciences [1,16], extending from the biological subspecialties of epidemiology through social and environmental epidemiology and into the social sciences – widely defined to include such fields as psychology, sociology, anthropology and economics, thereby providing a range of insights into the determinants of both individual and collective behaviour – and the environmental sciences (Table 5) [26-31].

The Centers for Disease Control and Prevention have defined translational research in public health as being concerned with institutionalising 'proven', 'evidence-based public health interventions' [32]. Our framework recognises that it is unrealistic always to expect or require unequivocal evidence of effectiveness of this kind – such as might be required for the introduction of new drugs – before recommending or advocating action to improve or safeguard public health (Table 4) [33]. While it is clearly essential to gather high-quality evidence about the effects of interventions, the recent Foresight project on obesity has illustrated how other types of evidence may influence public health action, for example by demonstrating adverse trends in health-related behaviour or by identifying environmental correlates of behaviour that could form the basis for interventions (Table 6) [34,35]. Intervention studies can also contribute crucial knowledge other than that derived from efficacy or effectiveness data, such as qualitative insights into participants' motivations and experiences.

All three domains of public health research identified in the Frankel report [16] – understanding causes, identifying strategies and evaluating their impact – are represented at multiple, overlapping points in the framework. If 'understanding causes' is taken to be synonymous with 'basic sciences', then the basic sciences of public health are seen to have roles all over the framework – for example in understanding the influence of the media on behaviour or on policymaking, or in developing and validating instruments for use in population health surveillance – and not merely in providing the 'evidence' that is to be implemented. 'Policy' is seen as both another class of interventions whose impact on the population require to be examined, and another class of causes whose influences on health-related behaviour require to be examined by these basic sciences (Table 3) [36].

#### An expanded role for evidence synthesis

Our framework identifies a pivotal role for evidence synthesis – albeit of a more inclusive nature than is necessarily typical of most current systematic reviews, which are often exclusively concerned with evidence of efficacy or effectiveness. At the same time, it acknowledges that isolated research findings or even 'factoids' (assertions repeated so frequently that they are assumed to be true) influence policy and practice, whether helpfully or unhelpfully (Table 5) [37-40]. This reflects an unresolved tension between the apparently uncontentious principle that the findings of single research studies should be disseminated to groups of key policymakers and practitioners and an alternative view that it may be inappropriate or even harmful to do so without placing those findings in the context of all other available findings on the same question [39,41,42] – a view reflected in the CONSORT statement on the reporting of randomised controlled trials [43].

It is increasingly argued – and often obvious – that evidence synthesis which embraces inputs from the wider range of disciplines and study designs outlined under the 'social perspective' above may be unlikely to produce hard-edged findings that can easily be translated into hard-edged recommendations for practice in terms of 'what works' [44,45]. To the more subtle contextual questions that are often important to decision-makers [46] – such as those posed by realist evaluation: 'What works, for whom, in what circumstances?' [47] – we might add that evidence synthesis may produce pointers to unintended or inequitable effects [48]. The more complex and nuanced nature of these outputs suggests a need for a feedback loop from the findings of evidence synthesis that triggers the refinement of intervention strategies and intervention hypotheses and their retesting using the most appropriate study designs [49]. It also raises questions about how the findings might be translated into practice (Table 6).

#### A non-linear interface with the real world

The implied linear, rational way in which new knowledge is converted into practice in the translational medicine pathway has limited empirical support or practical utility [14,50,51] and in any case, the practice that requires to be influenced is not limited to clinical practice or even public health practice. The targets for change in, for example, attitudes and behaviour may include practitioners working in other sectors (e.g. planners, architects, teachers and caterers), civil servants, industry, politicians, and opinion and culture in the population at large, for example through the media (Table 3, Table 5 and Table 6). We have labelled these wider targets for influence as the 'public realm' – a realm in which opinion-formers and decision-makers are unlikely to wait until researchers have satisfied themselves as to what constitute 'evidence-based public health interventions' [24]. On the contrary, our framework reflects an understanding that they may be influenced by more complex and non-linear pathways such as that described in the 'enlightenment' or 'limestone' model of the gradual sedimentation of ideas in the minds of research users [52] – an example of the 'indirect insinuation' of evidence into policy [53,54].

Moreover, interventions are rarely developed and introduced only as a result of public health (or any other) research. Many interventions that may influence health are introduced for other reasons. These include: a perceived political need to 'do something' in the absence of strong scientific evidence that the chosen response is likely to be effective [55] – possibly as a reaction to surveillance data, to a 'factoid' [40], or to more robust but 'non-causal' evidence about correlates of behaviour; the application of the precautionary approach; or to address goals in another sector such as those of transport, environmental or educational policy. Our framework therefore also reflects these other drivers that may lead to (or prevent) the introduction of interventions and also, therefore, the opportunity to study their effects. It shows that health policy is only a subset of the policy arena that may be the target of the knowledge produced by public health research and may give rise to interventions that influence health (Table 6).

### Implications for translational research

The framework suggests a considerable programme of work to characterise and operationalise the elements of the framework, populate them with evidence and translate that evidence into population health improvement. This amounts to a translational agenda for public health research.

#### Descriptive

The first area of work would involve describing the various elements and links and how they are related, for example by identifying the critical interfaces, receptors and processes for translation [52] and barriers to translation [56] at each link. It would also involve reviewing the theoretical understanding of how the processes considered important at each link are supposed to work, as exemplified by current work on the theoretical basis of behaviour change in public health interventions [57].

#### Effectiveness

The second area of work would involve reviewing the evidence as to which processes are effective in practice and identifying what further research would contribute most to the understanding and effectiveness of each link. An extensive literature on knowledge translation has already demonstrated the effectiveness of practices such as interactive engagement compared with disseminating materials for passive consumption [58]. However, knowledge translation in these terms forms only one element of our framework; different literature, or new primary research, may be required to identify effective strategies required elsewhere in the framework. For example, how can public opinion be influenced in support of a given intervention, how can authors and journals ensure that intervention studies are reported with sufficient detail about implementation to inform subsequent evidence synthesis [59], or how can the results of evidence synthesis be effectively translated into new, more useful intervention studies?

#### Operationalisation

The third area of work would involve characterising the roles and processes by which the various elements and links are (or could be) operationalised. Although the literature on knowledge translation supports the need for credible intermediaries [58], the diversity of terms currently in circulation – such as 'research brokers', 'research translators' and 'translational scientists' – illustrates the need for operational clarity. This could be elaborated by identifying the potential contributions of different disciplines such as epidemiology, economics and sociology, for example by building on the illustrative 'transdisciplinary/translation matrix' described by Sussman and colleagues [60]. It would also involve disaggregating elements currently represented as somewhat abstract 'black boxes' in the framework. To give two examples, the analysis of the spectrum of modifiable determinants of health could be disaggregated into the analysis of the relationship between exposure and outcome (aetiological epidemiology) and the analysis of the determinants of exposure that could form the basis for interventions, and the newly revised Medical Research Council (MRC) framework for complex interventions provides an obvious starting point for operationalising the development of a spectrum of possible interventions from theory and modelling through to long-term implementation [61,62].

#### Strategic

The fourth area of work would involve reflection and debate on the evidence gathered to agree where research and operational effort should be concentrated to achieve maximum translational impact. For example, Lavis has argued that the 'natural unit' for research translation should be 'actionable messages' arising from systematic reviews, and that the effort of promoting research findings to a given category of user should be concentrated on the fraction of systematic reviews that have an actionable message for that particular audience [63]. This suggests that undertaking *a priori *to 'disseminate' the findings of a particular piece of public health research in the public domain (as opposed to making the findings visible to others working within the overall translation framework) might, in some cases, be inefficient or even harmful.

### Implications for research funding

Our framework illustrates Cooksey's observation that public health research cannot readily be classified as basic or applied, and indeed goes further by showing how a wide range of public health research may contribute to the overall translational framework. Even where a given piece of research does not, or should not, give rise to an 'actionable message' for policymakers, practitioners or the general public, it may nonetheless contribute a crucial piece to the jigsaw by which the sum of public health research is eventually translated into population health improvement.

The first implication for research funders is that resources for 'applied' or 'translational' public health research should be deployed across the framework and not be reserved for a subset of studies concerned with 'dissemination' or 'implementation' of 'evidence-based interventions'. The second is that in systems for appraising the quality of research, the translational significance or performance of a given piece of research should be assessed in terms that realistically reflect its position within the framework: for some studies, an appropriate objective may be simply to inform debate, whereas for others, it may be to stimulate better or different research or to improve the methods by which others achieve those objectives [58].

## Summary

Despite the ambitious aims of public health reflected in the Acheson definition, the public health research community cannot expect simply to issue pronouncements that are accepted without question and result in the transformation of society [64]. The influential paradigm of translational medicine provides a useful starting point for considering the translation of public health research, but does not adequately reflect the complex reality of the public health environment. We have therefore outlined a translational framework for public health research, identifying some gaps in knowledge and practice and enumerating a research agenda to be pursued.

All types of public health evidence, 'from epidemiology to evaluation', may contribute to the shaping of policy, practice and future research, particularly when combined using thoughtful approaches to evidence synthesis. The framework and case studies show that further research is required to understand, populate and operationalise all the elements in the framework. It is not simply a matter of applying what we already know; the 'basic sciences' of public health still have much to contribute throughout the framework. At the same time, rigorous evaluative and implementation research is increasingly required and should not be regarded as inferior to the more traditional public health sciences.

## Competing interests

All the authors are employed by the MRC. Between them they hold, or have held, research grants from, and have served as members of grant-awarding panels for, a range of organisations including the MRC and other UK research councils, the UK Clinical Research Collaboration, the National Institute for Health Research and the Department of Health. PC is also employed by the Chief Scientist Office of the Scottish Government Health Directorates. The authors' employers had no role in the development of the framework or the writing of the paper. The corresponding author had final responsibility for the decision to submit for publication.

## Authors' contributions

The idea of developing a translational framework for public health research was developed in board meetings of the Population Health Sciences Research Network of which SM is chair, NW is a member and PC is the programme manager. DO reviewed the literature, developed the framework and wrote the paper. All authors contributed to the conceptual development of the paper and the critical revision of the manuscript and approved the final version.

## Pre-publication history

The pre-publication history for this paper can be accessed here:


